# The Use of 1α,25-Dihydroxyvitamin D_3_ as an Anticancer Agent

**DOI:** 10.3390/ijms17050729

**Published:** 2016-05-13

**Authors:** Ewa Marcinkowska, Graham R. Wallace, Geoffrey Brown

**Affiliations:** 1 Laboratory of Protein Biochemistry, Faculty of Biotechnology, University of Wroclaw, Joliot-Curie 14a, 50-383 Wroclaw, Poland; ema@cs.uni.wroc.pl; 2 Institute of Inflammation and Aging, College of Medical and Dental Sciences, University of Birmingham, Edgbaston, Birmingham B15 2TT, UK; g.r.wallace@bham.ac.uk; 3 Institute of Clinical Sciences, College of Medical and Dental Sciences, University of Birmingham, Edgbaston, Birmingham B15 2TT, UK

**Keywords:** cancer, 1α,25-dihydroxyvitamin D, analogues

## Abstract

The notion that vitamin D can influence the incidence of cancer arose from epidemiological studies. The major source of vitamin D in the organism is skin production upon exposure to ultra violet-B. The very first observation of an inverse correlation between exposure of individuals to the sun and the likelihood of cancer was reported as early as 1941. In 1980, Garland and Garland hypothesised, from findings from epidemiological studies of patients in the US with colon cancer, that vitamin D produced in response to sun exposure is protective against cancer as opposed to sunlight *per se*. Later studies revealed inverse correlations between sun exposure and the occurrence of prostate and breast cancers. These observations prompted laboratory investigation of whether or not vitamin D had an effect on cancer cells. Vitamin D is not active against cancer cells, but the most active metabolite 1α,25-dihydroxyvitamin D_3_ (1,25D) has profound biological effects. Here, we review the anticancer action of 1,25D, clinical trials of 1,25D to date and the prospects of the future therapeutic use of new and low calcaemic analogues.

## 1. Introduction

Differentiation of mouse myeloid leukaemia cell line towards macrophage-like cells in response to a sub-nanomolar concentration of 1α,25-dihydroxyvitamin D_3_ (1,25D) was reported in 1981 [1]. The changes observed were striking; the leukemic cells exposed to 1,25D, not only looked different, but were also able to phagocytose, produce antibacterial agents, migrate and adhere to substratum [1]. A similar effect of 1,25D on human acute myeloid leukaemia (AML) cells was reported soon after [2]. In this case, changes in cell cycle progression were observed to accompany differentiation to monocytes. The changes were a transient increase in the proportion of cells in the G2/M phase of cell cycle, followed by an arrest in the G0/G1 phase [3]. In other words, 1,25D provides an initial signal for cell proliferation, followed by different signal leading to cell maturation and cycle arrest [4]. This complicated set of events, which is triggered by 1,25D, requires the coordinated actions of signalling molecules and transcription factors.

## 2. 1α,25-Dihydroxyvitamin D_3_-Driven Cell Signalling in Acute Myeloid Leukaemia (AML) Cells

It is widely accepted that expression of a functional vitamin D receptor (VDR) within AML cells is required for 1,25D to exert its biological effects [5]. In support of this viewpoint is that AML cells, which express VDR at a low level, are resistant to the differentiating effects of 1,25D, and the sensitivity of AML cells to 1,25D is restored when the level of VDR is upregulated [6]. That the presence of VDR is essential to the differentiating action of 1,25D was confirmed by the finding that normal blood progenitor cells from VDR knock-out mice failed to differentiate into monocytes in response to 1,25D [7].

VDR is a ligand-activated transcription factor that controls the transcription of hundreds of genes, either directly or indirectly [8]. Among the VDR-target genes are ones that encode the functional attributes of mature macrophages, such as cluster of differentiation (CD) 14 [9], and genes that control cell cycle progression are 1,25D responsive [10]. VDR protein is translated in the cytosol, and its site of transcriptional activity is the cell nucleus. Therefore, efficient nuclear translocation of VDR is prerequisite for its activity and AML cells in which nuclear transport of VDR is hampered do not differentiate when treated with 1,25D [11]. AML-associated chromosome translocation products, such as promyelocytic leukaemia (PML)/retinoic acid receptor α (RARα), promyelocytic leukaemia zinc finger protein (PLZF)/RARα, and AML-1/eight-twenty one protein (ETO), may block transport of VDR to the nucleus and reduce the capacity of AML cells to differentiate in response to 1,25D [12].

However, activation of VDR’s transcriptional activity is not sufficient to explain all of the actions of 1,25D against AML cells. Hours are required for 1,25D to result in VDR-mediated transcription of gene products, and some of the biological effects of 1,25D observed within AML cells happen in minutes. As to immediate effects in AML cells, 1,25D activates protein kinase C (PKC) [13], the extracellular-signal activated kinases (Erk) 1 and 2 [14] and the phosphatidyl inositol 3-kinase (PI3K) [15]. Additionally, 1,25D activates lipid-signalling pathways in AML cells. These are important to 1,25D-driven cell differentiation as it has been shown that inhibition of phospholipase D (PLD) blocks 1,25D-induced AML cells differentiation [16]. Enzymatic activity of PLD leads to the breakdown of membrane phospholipids, such as phosphatidylcholine and produces a second messenger, diacylglycerol (DAG). DAG in turn is an activator of the classical isoforms of PKC, which have been shown to be rapidly activated by 1,25D [13] and are on the pathway leading to activation of Erk1,2 [14]. Another lipid signalling enzyme that is activated in AML cells in response to 1,25D is phospholipase A_2_ (PLA2) [17]. Activation of PLA2 starts as early as three hours and lasts at least for 48 h post-exposure of cells to 1,25D. Experiments using inhibitors of PLA2 have shown that 1,25D-induced differentiation is enhanced when the activity of PLA2 is inhibited. These experiments suggested that the end products of the PLA2 metabolic pathway provide a negative feedback to the action of 1,25D [18,19].

The mechanism, whereby immediate signal events are provoked, is still not entirely clear [20] as a number of mechanisms have been proposed to explain this phenomenon. One hypothesis is that a small portion of classical VDR is localized at the plasma membrane and 1,25D binds to an alternative ligand binding pocket to activate signal transduction from the cell membrane [21]. An alternative viewpoint is the existence of a membrane receptor for 1,25D that is distinct from VDR and is responsible for the rapid actions of 1,25D. Such a receptor has been shown to be expressed by intestine cells and is termed Membrane Associated Rapid Response Steroid-binding (MARRS) protein, which is also known as ERp57 or protein disulfide-isomerase A3 (PDIA3) [22,23]. Whether this receptor is present in AML cells has not been determined.

A primary feature of AML cells is their uncontrolled proliferation. In patients, the leukemic blast cells outnumber other blood cells. Moreover, the cells accumulating are non-functional because of a block at an early stage of myeloid cell differentiation. The use of 1,25D to inhibit the proliferation of AML cells and drive differentiation of these cells towards macrophages would be beneficial to patients or, at least, make their disease bearable. Additionally, AML patients are deficient in innate immune responsiveness and are prone to infection; restoration of some macrophage-related immunity might be beneficial. Unfortunately, there is a major drawback to the use of 1,25D as an anticancer agent and achievement of an effective therapeutic dose. The primary role of 1,25D is to regulate calcium and phosphate homeostasis, and when 1,25D is administered to patients at a high dose there are undesirable calcaemic effects. Serious side effects arising from hypercalcaemia include coma and cardiac arrest. Intermittent use of a high dose of 1,25D in patients with advanced prostate cancer has been reported to be safe. However, the addition of 1,25D to dexamethasone treatment did not significantly improve the response rate [24]. The principle strategy to overcoming the problem of calcaemic effects has been the development of analogues of 1,25D with the desired anticancer effect without being calcaemic. Numerous analogues of 1,25D that have negligible calcaemic action have been synthesized [25,26]. Interestingly, neither the calcaemic nor the anti-proliferative potentials of analogues of 1,25D analogues can be correlated directly to their affinities to VDR [26].

## 3. The Actions of 1α,25-Dihydroxyvitamin D_3_ against Carcinomas

The hypothesis that vitamin D deficiency is a risk factor in terms of the development of prostate cancer was proposed in 1990 [27,28] and was based on a former hypothesis from 1941 [29]. Schwartz and Hulka proposed that an appropriate level of vitamin D is essential to maintain the normal phenotype of prostate cells. Soon after, investigators documented the presence of VDR in prostate cells [30]. Later, epidemiological studies showed that serum levels of 25-hydroxyvitamin D_3_ (25D) adversely correlate with prostate cancer risk [31]. This was most obvious in men living at high latitude, for example, in Scandinavia, whose 25D level in blood serum was very low, below 16 ng/mL [32]. Numerous publications followed, which documented that 1,25D has a beneficial action against prostate cancer cells. 1,25D was observed to inhibit proliferation, stimulate apoptosis, and interestingly have an anti-inflammatory effect towards VDR-positive prostate cancer cells [33]. These influences were accompanied by a decrease in the capacity of prostate cancer cells to migrate and, accordingly, their invasive potential [34,35]. A most surprising observation was that prostate cells have the capacity to convert biologically inert 25D into biologically active 1,25D. Previously, the only organ that was thought to be able to hydroxylate 25D to 1,25D was the kidney. In 1998, it was shown that normal prostate cells also possess the 1-α-hydroxylase to 25D (CYP27B1), which provides prostate cells with the means to synthesize 1,25D [36]. The activity of CYP27B1 has been examined in relation to the progressive steps of prostatic neoplasia, and the activity of CYP27B1 diminishes as cells progress from a normal phenotype, through hyperplasia, to a malignant phenotype [37]. Other studies have provided data to further support the notion that a correct level of vitamin D and its autocrine conversion to 1,25D in the prostate are protective factors in prostate neoplasia. A high dietary intake of calcium has also been found to correlate with prostate cancer incidence [38] and this has substantially complicated the picture as a high level of circulating 1,25D increases the uptake of calcium from the intestine. An understanding of the role of 1,25D in prostate neoplasia is, as yet, incomplete but that 1,25D plays an important role in the development of prostate and prostate cancer is not questioned.

A large proportion of breast cancer cells contain the VDR protein, however the level of expression is variable within individual cells [39] and, therefore, the biological response to 1,25D varies for an individual patient’s cells and between patients. The primary effect of exposure of breast cancer cells to 1,25D is cell cycle arrest due to changes in the functional status of the proteins that regulate cell cycle, such as retinoblastoma protein (Rb) and cyclin-dependent kinases and their inhibitors [40]. The induction of apoptosis is mediated by a reduction in the level of bcl-2 and up-regulation of the level of p53 [41]. An effect of 1,25D on cell proliferation is also mediated in a secondary way, via interference to the function of oestrogen receptors (ER). 1,25D and its analogues down-modulate the expression of ERα, which in turn reduces the level of mitogenic signals to breast cancer cells from oestrogens [42]. Another mechanism of the anticancer action of 1,25D against breast cancer cells is that it down-regulates the expression of aromatase, which catalyses a step in oestrogen synthesis [43]. One of the key factors in regard to the activity of 1,25D is its availability in the breast cancer environment. This is maintained by the balance between synthesis and catabolism. CYP27B1 is present in some breast cancer cells, to control the autocrine synthesis of 1,25D, but this enzyme is also active in breast cancer microenvironment. It has been shown that breast adipocytes produce CYP27B1, bio-activate 25D to 1,25D and in a paracrine fashion deliver 1,25D to the breast epithelium [44]. On the other hand, the availability of 1,25D is maintained by its degradation. This process is maintained by the 24-hydroxylase of 1,25D (CYP24A1). In normal tissues, this enzyme is expressed in response to 1,25D exposure, providing a regulatory mechanism that maintains the concentration of 1,25D at a desired level [45]. Genome hybridization studies have revealed that in certain human breast cancers the *CYP24A1* gene is amplified and this may cause a reduction in the level of 1,25D level and cells to proliferate unduly [46].

As mentioned above, an inverse correlation between the incidence malignancy and the level of vitamin D was first observed for colon cancer [47]. *In vitro* studies have shown that 1,25D and its analogues are able to inhibit the proliferation of colon cancer cells [48]. The anti-proliferative effect of 1,25D is revealed by an accumulation of cells in the G0/G1 phase of the cell cycle, and this is caused by an enhanced expression of cyclin-dependent kinase inhibitors p21^CIP1^ and p27^KIP1^ and reduced expression of cyclin A and cyclin F [49]. In addition, 1,25D sensitizes colon cancer cells to pro-apoptotic signals, through up-regulation of the pro-apoptotic and the down-regulation of the anti-apoptotic proteins [49]. Many studies have revealed that 1,25D promotes differentiation of colon cancer cells. This has been measured in terms of an expression of brush border enzymes and adhesion molecules, which confer the proper structure of colon epithelium, and acquisition of phenotypic features such as the presence of microvilli and cells having a polarized structure [49]. As for breast cancer, the vitamin D metabolic pathways appear to be important to colon tumorigenesis. *CYP27B1* expression is enhanced in high- to medium-differentiated human colon tumours as compared to tumour-adjacent normal mucosa or colon mucosa from non-cancer patients. In high-grade undifferentiated tumour areas expression of *CYP27B1* is decreased [50]. In contrast and in the majority of colon adenocarcinomas, expression of the 1,25D degrading enzyme CYP24A1 is enhanced [51]. Moreover, the *CYP24A1* gene locus has been shown to be amplified in the majority of colorectal cancers and this gene has been proposed to be an oncogene [52].

## 4. 1α,25-Dihydroxyvitamin D_3_ and Anticancer Immunity

1,25D may be used as an anticancer agent alone and in combination with chemotherapeutic drugs as a means of enhancing the potency of such. As to the latter use, patients receiving chemotherapy are highly vulnerable to infections, which can lead to death. Therefore it is important to ensure that 1,25D and new analogues are beneficial to good health and particularly do not have a damaging effect on the patient’s immune system. In essence, an appropriate level of 1,25D is essential for good health and as to the correct function of the innate and adaptive immune systems.

The ancient Greeks first observed the benefit of exposure to sunlight, and presumably the synthesis of 1,25D, to good health. They used heliotherapy to treat the disease we now know as tuberculosis (TB). In 1903, Niels Finsen won the Nobel Prize for showing that exposure to ultra-violet light could be used to treat cutaneous TB. The need to promote public awareness of the importance of vitamin D to good health stems from knowledge of the consequences of vitamin D deficiency. A low systemic level of 1,25D leads to a higher risk of chronic diseases that affect a variety of organs. Such include diabetes [53], hypertension [54], inflammatory bowel disease [55], and kidney disease with a poor outcome at late stage [56]. Vitamin D also protects against osteoporosis [57], delays the onset of type 1- and 2-diabetes [53] and lowers the blood pressure in hypertensive animals [58]. There is the possibility that the association studies are revealing that 25D is a marker of poor health/lifestyle in general. Even so, cod liver oil has been used for over 100 years as an excellent source of vitamin D and a cure-all supplement, and the American Institute of Medicine recommends adequate intake (from UV, diet or supplement) of vitamin D at 600 IU per day up to the age of 70 and 800 IU if older [59].

1,25D enhances the protective effects of the innate immune response. Individuals who are vitamin D deficient [60] and those with inactivating mutations in the gene encoding VDR show defects in responses towards infectious agents [61]. Tiosano and colleagues examined the immune status of fifteen hereditary vitamin-D resistant patients that have a truncated VDR, which is unable to bind 1,25D and devoid of function. They reported no increased incidence of infectious, or autoimmune, diseases, but there were some differences between people with wild type and mutated VDR as to their capacity to mount immune responses [62]. In keeping, it has been suggested that supplementation of vitamin D can help to fight off viral and other infections by boosting the function of the immune system [63,64]. Most of the cells of the immune system express VDR and these cells also have the enzymes to make 1,25D from circulating 25D. As such, locally produced 1,25D plays an important role in enhancing the status of innate immunity. Blood-derived macrophages express VDR and can make their own 1,25D [65]. 1,25D promotes the proliferation and maturation of monocytes and macrophages and the migration of these cells to sites of infection. At sites of infection, the recognition of antigens by macrophage pattern recognition receptors switches on the production of 1,25D which, in turn, stimulates phagocytosis and the expression of the antimicrobial peptides defensin β2 and cathelicidin gene product, to increase microbial killing (reviewed in [66]). However, production of AMP may not be the only mechanism by which 1,25D controls infection. In a murine model of pulmonary TB, bacterial load was unaffected, but granuloma size was increased and interferon γ (IFNγ) and tumour necrosis factor (TNF) producing CD4 cells decreased in animals on a high vitamin D diet. The reduction in immunopathology was protective against the pathogen and to the lung tissue [67]. Antimicrobial peptides, such as cathelicidin, have been implicated in both inducing, via receptors such as formyl receptor 2 (FRP2) and cell proliferation, and preventing cancer via direct binding to negatively charged membranes of tumour cells and induction of apotosis [68,69].

1,25D may be of particular benefit to the immune status of older patients with cancer. With age, the immune system senesces and shifts towards an inflammatory profile, termed inflammaging. The chronic production of inflammatory cytokines appears to remodel the immune system [70,71]. Consequently, auto-immune diseases, such as rheumatoid arthritis, are more prevalent in older people [72]. In these disorders B lymphocytes make auto-antibodies with the help of T lymphocytes. 1,25D can dampen down the activity of the adaptive immune system. For example, *in vitro* studies have shown that 1,25D inhibits the generation of plasma cells, their expression of co-stimulatory molecules and the function of regulatory B cells. However, administration of a high dose of vitamin D to patients with multiple sclerosis has been shown to not have a substantial effect on B cell differentiation, isotype switching or the level of B cell activating factor [73]. Differences between findings from *in vitro* and *in vivo* studies indicate a more complex scenario and have led to speculation that the role of germinal centres in plasma cell production and/or other vitamins and hormones interfere with vitamin D-driven effects on B cells (reviewed in [74]). Further work is warranted to unravel the subtly of the influence of 1,25D on the B cell compartments.

Resting T lymphocytes do not express VDR but do so after they are activated. 1,25D has been shown to inhibit T lymphocyte proliferation and T helper (Th) cells that make interleukin (IL)-17 (Th 17 cells) and interferon-γ (Th 1 cells). Th 1 and Th 17 cells can mediate immune diseases and these diseases are ameliorated by treatment with 1,25D. Th 1 and Th 17 responses are also important to host resistance to infectious diseases; 1,25D does not affect these responses. These findings lead to the viewpoint that 1,25D is a late regulator of the function of T lymphocytes and this function is important when there is chronic activation of T lymphocytes (reviewed in [75]). As to switching off/controlling immune responses, 1,25D helps to maintain self-tolerance by skewing the maturation of CD4 T lymphocytes away from a Th1 phenotype towards a tolerogenic Th2 or regulatory T cell phenotype. In this case, secretion of interferon-γ, IL-17, IL-21 and IL-22 is suppressed and expression of forkhead box P3 (FOXP3), cytotoxic T-lymphocyte-associated protein 4 (CTLA-4) and IL-10 is enhanced. Importantly, a low-calcaemic analogue of 1,25D has been used to treat T cells from healthy individuals to promote a stable regulatory profile [76]. Dendritic cells, through their interaction with T lymphocytes, are responsible for the generation of adaptive immunity and also have a tolerogenic role. The presence of 1,25D leads to dendritic cells acquiring tolerogenic properties [77]. The maturation of these cells is repressed by 1,25D as to reduced expression of the major histocompatibility complex and the costimulatory molecules CD40, CD80 and CD86. Secretion of IL-12 is decreased and IL-10 expression is enhanced which contributes to dendritic cells becoming tolerogenic in nature (reviewed in [66,78]). The inhibition of T cell priming by dendritic cells by 1,25D may be more complex than currently considered. On activation, dendritic cells express a truncated *CYP27B1* transcript that inhibits the conversion of 25D to active 1,25D, while *CYP24A1* levels were unaffected, leading to reduced autocrine 1,25D. Activation of vitamin D to 1,25D by macrophages has no such restraint. The production of 1,25D by macrophages induces expression of VDR- responsive genes by dendritic cells and inhibits antigen stimulation-driven maturation of dendritic cells and dendritic cell-dependent T cell responses. These data reveal that autocrine and paracrine activation of vitamin D are important to the control of dendritic cell function. [79]. Recent evidence shows that 1,25D may not directly inhibit the generation of antigen-specific CD4 T cells. In animals with experimental autoimmune encephalitis, myelin specific CD4 cells in the blood numbers were not affected, but treated animals had significantly decreased disease scores. Such antigen-specific cells left the lymph nodes and were present in the circulation but they did not enter the brain parenchyma. This was shown to be due to down-regulation of CXCR3, a receptor required for entry into the central nervous system (CNS) tissue [80].

Studies of VDR knock-out mice have brought to attention that responsiveness of cytotoxic T lymphocytes (CTLs) might be somehow affected by the absence of signals from 1,25D and VDR. These mice were able to clear *Listeria monocytogenes* infection, but slower than wild type counterparts, and their CTLs produced less IFNγ [81]. These observations have focused attention on the role of 1,25D and VDR in CTL differentiation and cytokine production. A clear picture has still to emerge though the link between 1,25D and the incidence and/or severity of CTL-controlled infections is not disputable [60].

As stated above, low levels of 25D or 1,25D have been associated with disease in many autoimmune conditions. However, supplementation studies to date have proved inconclusive as to the prevention or control of autoimmune conditions, but this may be due to the dose and type of vitamin D used in the various studies [82]. A prospective randomised control trial with high dose vitamin D (up to 40,000 IU daily until 28 weeks, then 10,000 IU daily for 12 weeks) in patients with multiple sclerosis was safe and reduced the relapse rate at 52 weeks [83]. More such randomised control trials with increased doses are needed to confirm these findings.

Many of the above findings as to effects of 1,25D on immune cells indicate the potential of 1,25D to restore immune tolerance and prevent the progression of autoimmune disease. The immune system of elderly patients with cancer might well be strengthened by the use of 1,25D to enhance the maintenance of tolerance, leading to the avoidance of autoimmune disorders. A conceivable downside to the use of 1,25D to treat patients with cancer is whether any existing immunity to the tumour is switched from responsiveness towards a state of tolerance of the tumour cells. The extent to which this might occur is unknown and a benefit is most likely as to the anticancer action of 1,25D.

## 5. Clinical Trials of the Use of 1α,25-Dihydroxyvitamin D_3_ in Cancer

### 5.1. Myelodysplasia (MDS) and AML

The results from *in vitro* studies of the activity of 1,25D against AML cell lines [2], patient-derived AML blasts [84,85] and pre-clinical tests in mice [86,87] clearly encouraged clinical trials. However, neither vitamin D nor its partially or fully active metabolites have proved to be very effective in clinical trials. One of the reasons for this failure was the clinical trials were performed on small groups of patients, ranging from 1 to 53, and the groups consisted of just MDS or AML patients, or patients with both conditions were included [88,89,90,91,92,93,94,95,96,97]. Moreover, the vitamin D compounds used in the clinical trials varied, as some used 1,25D whilst other trials made use of the 1,25D precursor 1α-hydroxyvitamin D (1D, alfacalcidol). The doses used also varied, as well as the additions of other agents used in the treatments. The best results were observed for combination treatments. When MDS patients were treated with 1,25D and 13-*cis*-retinoic acid, the response rate was 52% and the transfusion need was reduced. However, the median survival time was not prolonged in these patients [96]. In a study that used 1,25D in combination with cytarabine and 13-*cis*-retinoic acid a response, to variable extent, was observed in 58% of patients [97]. Even better results were obtained using a combination of 1,25D with cytarabine and hydroxyurea: complete or partial responses were observed in 79% of elderly patients with AML [95].

### 5.2. Prostate Cancer

There were several early clinical trials of the use of vitamin D compounds to treat prostate cancer. A pilot study revealed that administration of 2000 IU of vitamin D daily led to a statistically significant delay in the rate of prostate specific antigen (PSA) rise in post-operative patients [98]. Another bigger study, conducted on 66 patients, confirmed the above results, and supported continuation of clinical trials [99]. In the later clinical study, performed on patients with advanced prostate cancer, 1,25D, the active form of vitamin D, was given together with dexamethasone [24]. Unfortunately, the response rate to this combination treatment was not seen to be significantly higher than when dexamethasone was given alone. However, high-dose intermittent 1,25D plus dexamethasone appeared to be safe for patients. The above, and some other encouraging results, led to the development of a formulation of oral high dose 1,25D, named DN-101, by Novacea Inc. (San Francisco, CA, USA). This new formulation in combination with docetaxel *versus* placebo and docetaxel was tested in a large, double-blinded, randomized study named ASCENT-1 [100]. The primary end-point, which was a PSA response within 6 months of enrolment, was not significantly different as to the DN-101 *versus* placebo arm. However, the median survival, which was not a primary endpoint in the trial, was estimated at 24.5 months in the DN-101 arm *versus* 16.4 months for placebo. This large survival advantage led to the next clinical trial, named ASCENT-2 [101]. Unfortunately, ASCENT-2 was terminated by Novacea Inc. on 5 November 2007, because of an unexpected and unexplained increased death rate in DN-101 arm.

Caution is warranted with respect to the use of vitamin D compounds to treat prostate cancer as the overall role of 1,25D in aggressive prostate cancer, in particular as to anti-inflammatory effects and disease pathogenesis and progression, is not well explored [102]. Additionally, it has been shown that men with high blood levels of 25D are at increased risk of developing prostate cancer [103].

### 5.3. Breast Cancer

Many *in vitro* and pre-clinical studies have examined the possible use of 1,25D to treat patients with breast cancer. Four meta-analyses identified a significant inverse relationship between the circulating concentrations of 25D and breast cancer [104,105,106,107]. By contrast, the large randomised clinical trial WHI showed that administering 400 IU vitamin D and 1000 mg of calcium *versus* placebo to women did not reduce the risk of breast cancer [108]. The finding from Women’s Health Initiative (WHI) does not provide support to the use of vitamin D as a prophylactic agent in the case of breast cancer. However, better survival among woman diagnosed with breast cancer has been reported to be related to vitamin D intake leading to higher concentrations of 25D [107,109]. Moreover, an interesting finding emerged from a retrospective review of patients with HER2^+ve^ breast cancer who had received chemotherapy with trastuzumab with or without vitamin D supplement (10,472 IU/week). Those who had taken the vitamin D supplements experienced significantly improved disease-free survival [110]. The conduct of a randomised, double-blind, placebo-controlled trial of vitamin D, or its active hormone 1,25D, with chemotherapy or other agents, is clearly important to revealing the benefit or otherwise of vitamin D/1,25D to treating breast cancer patients.

### 5.4. Colorectal Cancer

Colorectal cancer has been a particular area of focus as to epidemiological studies of the role of vitamin D in cancer (reviewed in [111]) and pre-clinical studies of the use of 1,25D. As to studies of patients with colorectal cancer, there is a high prevalence of vitamin D deficiency among patients with stage IV colorectal cancer [112] and other studies support the notions that the formation of new adenomas appears not to be inhibited by vitamin D though progression through the carcinogenesis pathway and the growth of lesions are influenced by vitamin D [113]. A trial of vitamin D and calcium in patients who had had their colorectal adenomas removed did not reduce the risk, over a period of three to five years, of adenomas recurring [114]. However, whether vitamin D and calcium can prevent the recurrence of colorectal adenoma is still under investigation in the large, double-blind and randomised clinical trial The Vitamin D/Calcium Polyp Prevention Study [115]. The pending results should provide further insight to whether vitamin D can reduce the incidence of adenoma recurrence. Hence, there is no definitive evidence from patient studies to support vitamin D/1,25D as a reliable means to prevent and/or treat colorectal cancer. As brought to attention in a recent article, data obtained from studies of colorectal cancer provides the most compelling evidence as to a beneficial relationship between intake of vitamin D, and the level of serum 25D, and cancer, but the promise is not yet fulfilled [116]. However, it is important to bear in mind there has been much discussion between vitamin D experts on what is a correct level of intake [117]. Even so, there has been a long-term interest in the use of vitamin D/1,25D to treat colorectal cancer and more controlled and randomised trials are needed to resolve this matter.

### 5.5. Melanoma

Eighty percent of the daily requirement of vitamin D is made by the skin depending on appropriate exposure to sunlight. Melanoma is the most dangerous type of skin cancer and a low serum concentration of 25D is associated with both an increased risk of melanoma and unfavourable disease prognosis [118]. Studies have suggested a role for vitamin D in delaying the recurrence of melanoma and there are few treatment options for patients with primary cutaneous melanomas that are ulcerated and patients with nodal micro-metastases. ANZMTG (Australia and New Zealand Melanoma Trials Group) 02.09 Mel-D is an on-going placebo controlled randomised phase II trial that is examining the safety and toxicity of an initial oral and large dose of vitamin D (500,000 IU) followed by a monthly oral dose of 50,000 IU for two years in patients who have had surgery to excise their primary cutaneous melanoma [119]. The findings from this study will be of interest as to the possible use of vitamin D, given orally, to treat another hard-to-treat cancer.

## 6. The Extent to Which Low Calcaemic Analogues Are in Use

Recently, the result of a phase I multicenter trial in metastatic prostate cancer patients which combined the 1,25D analogue inecalcitol with docetaxel-based chemotherapy has been published. Inecalcitol is an analogue of 1,25D that shows agonistic activity towards VDR and was well tolerated in preclinical studies in mice [120]. The phase I study documented that inecalcitol was well tolerated and that 85% of patients had more than a 30% decline in PSA level within three months, while 76% of the patients had more than a 50% PSA decline at any time during the study [121].

Another analogue of 1,25D is paricalcitol, which is related in chemical structure to vitamin D_2_. This analogue was tested in women receiving chemotherapy for metastatic breast cancer and the study documented that paricalcitol in combination with taxanes is safe and feasible [122]. This opens the possibility of larger clinical trials to assess health benefits of this analogue in women with breast cancer.

## 7. Conclusions

We can conclude there has not been substantial benefit to date as to the use of 1,25D to treat cancers. The results of trials conducted so far show that neither 1,25D nor analogues are sufficient anti-cancer agents when used alone. In essence, response rates have been insufficient and variable and there have not been any dramatic increases in survival rates. *In vitro* and preclinical studies support the viewpoint that the various types of cancer are somewhat equally susceptible to the effects of 1,25D [123], and epidemiological studies clearly indicate that a correct level of serum vitamin D correlates with a low cancer incidence [111]. Careful analysis of these results may indicate that vitamin D is one of a few factors that correlate with the prevention of carcinogenesis. However, supplementing one of the factors, just vitamin D, may be not enough to slow down or cure the disease. Therefore, studies that aim to find accompanying factors, which act in concert with vitamin D, are necessary. Another possibility is that a low level of vitamin D or 25D is not causative for the above-mentioned diseases. It should be also considered that such is a marker of poor health or a bad lifestyle. As yet, the verdict is uncertain in regard to the use of 1,25D analogues as an anticancer agent particularly as the new analogues with negligible calcaemic action offer exciting prospects. These offer the possibility of achieving a more effective therapeutic dose with minimal side effects. When used in combination treatments they may deliver enhancement of the activities of other anti-cancer drugs or health benefits from their immune-modulatory actions.

## Abbreviations

1,25D	1α,25-Dihydroxyvitamin D_3_
25D	25-Hydroxyvitamin D_3_
AML	Acute myeloid leukaemia
CD	Cluster of differentiation
CNS	Central nervous system
CTL	Cytotoxic T lymphocyte
CTLA-4	Cytotoxic T-lymphocyte-associated protein 4
CYP24A1	24-Hydroxylase of 1,25D
CYP27B1	1-α-Hydroxylase to 25D
DAG	Diacylglycerol
ER	Estrogen receptors
Erk	Extracellular-signal activated kinase
ETO	Eight-twenty one protein
FOXP3	Forkhead box P3
FRP2	Formyl receptor 2
IL	Interleukin
IFNγ	Interferon γ
MARRS	Membrane Associated Rapid Response Steroid-binding
MDS	Myelodysplasia
PDIA3	Protein disulfide-isomerase A3
PI3K	Phosphatidyl inositol 3-kinase
PKC	Protein kinase C
PLA2	Phospholipase A_2_
PLD	Phospholipase D
PLZF	Promyelocytic leukaemia zinc finger protein
PML	Promyelocytic leukaemia
PSA	Prostate specific antigen
RARα	Retinoic acid receptor α
Rb	Retinoblastoma protein
Th	T helper
TNF	Tumour necrosis factor
VDR	Vitamin D receptor
WHI	WOMEN’S Health Initiative
